# WELL-BEING AND FUNCTIONING PROFILE AMONG OLDER ADULTS BETWEEN DIFFERENT LIVING SETTINGS

**DOI:** 10.1093/geroni/igae098.3087

**Published:** 2024-12-31

**Authors:** Wan-Ling Hsu, SangNam Ahn

**Affiliations:** Saint Louis University, St. Louis, Missouri, United States; Saint Louis University, St. Louis, Missouri, United States

## Abstract

Addressing the care needs of older adults in the United States is complex and challenging, especially considering that more than half of Americans aged 65 years and older have three or more chronic diseases, often accompanied by cognitive impairment. Thus, it is imperative to identify the types of care that may be more suitable for older adults who are potentially cognitively impaired. This study examined well-being and functioning profile (WFP) among older adults with probable or possible dementia between nursing home and comprehensive senior living settings, using the living settings as a proxy for types of care. The WFP includes assessments of emotional well-being, bothersome pain, general health, and functional limitations. The analysis is based on data collected from the National Health and Aging Trends Study spanning from 2018 to 2022. Linear regression with survey weights revealed a significant association between living settings and WFP among the 338 participants with probable or possible dementia. Specifically, older adults residing in comprehensive senior living settings exhibited notably enhanced emotional well-being (Coef= -5.276, p < 0.001), general health (Coef= -1.117, p = 0.006), and limitations with mobility (Coef=-3.180, p = 0.003) compared to those in nursing home settings. Findings emphasize the potential strength of comprehensive senior living settings in caring for older adults with probable or possible dementia, enhancing improved outcomes. This prompts further research into specific care components and strategies to integrate successful practices from comprehensive senior living settings into nursing home settings, fostering well-being for this growing demographic facing cognitive challenges.

